# Diagnosis of forme fruste keratoconus with scheimpflug photography in Ghanaian patients

**DOI:** 10.1186/s12886-024-03563-x

**Published:** 2024-07-15

**Authors:** Seth Lartey, Emmanuel Appiagyei

**Affiliations:** 1 Eye Ear Nose and Throat Department, Kwame Nkrumah University of Science and Technology, Kumasi, Ghana; 2 Eye Department, Komfo Anokye Teaching Hospital, Kumasi, Ghana; 3 Department of Optometry and Visual Science, Kwame Nkrumah University of Science and Technology, Kumasi, Ghana

**Keywords:** Keratoconus, Forme fruste keratoconus, Pentacam, Diagnostic model, Sensitivity, Specificity

## Abstract

**Aim:**

This study aimed to differentiate moderate to high myopic astigmatism from forme fruste keratoconus using Pentacam parameters and develop a predictive model for early keratoconus detection.

**Methods:**

We retrospectively analysed 196 eyes from 105 patients and compared Pentacam variables between myopic astigmatism (156 eyes) and forme fruste keratoconus (40 eyes) groups. Receiver operating characteristic curve analysis was used to determine the optimal cut-off values, and a logistic regression model was used to refine the diagnostic accuracy.

**Results:**

Statistically significant differences were observed in most Pentacam variables between the groups (*p* < 0.05). Parameters such as the Index of Surface Variance (ISV), Keratoconus Index (KI), Belin/Ambrosio Deviation Display (BAD_D) and Back Elevation of the Thinnest Corneal Locale (B.Ele.Th) demonstrated promising discriminatory abilities, with BAD_D exhibiting the highest Area under the Curve. The logistic regression model achieved high sensitivity (92.5%), specificity (96.8%), accuracy (95.9%), and positive predictive value (88.1%).

**Conclusion:**

The simultaneous evaluation of BAD_D, ISV, B.Ele.Th, and KI aids in identifying forme fruste keratoconus cases. Optimal cut-off points demonstrate acceptable sensitivity and specificity, emphasizing their clinical utility pending further refinement and validation across diverse demographics.

## Introduction

Keratoconus is a non-inflammatory ectatic corneal dystrophy characterized by progressive corneal thinning that results in corneal steepening, protrusion, and irregular astigmatism [1]. While definite cases of keratoconus are identifiable through characteristic biomicroscopy and topographic findings, detecting subtle forms such as forme fruste or subclinical keratoconus has proven to be exceptionally difficult [2]. The term “forme fruste” denotes an early manifestation of the disease lacking overt keratometric, retinoscopic, or slit lamp indications but exhibiting mild topographic changes [3]. These cases may mimic symptoms of high myopia, astigmatism, or amblyopia, making their differentiation from other refractive errors a clinical challenge [4].

Recent advancements in diagnostic technologies have led to the integration of varied parameters derived from various imaging modalities, aiming to enhance the early detection of keratoconus. The Pentacam HR (Oculus Optikgeräte GmbH, Wetzlar, Germany) is an anterior segment tomography device, based on a rotating Scheimpflug camera. This technology provides significantly more information than anterior surface topography, as tomography utilizes data from the anterior and posterior surfaces of the cornea, as well as pachymetric mapping [5–8]. Early and advanced keratoconus detection using variable indices of the Pentacam has been widely discussed, and the sensitivity and specificity of various parameters have been compared [9–11].

Recent treatments such as corneal collagen cross-linking (CXL) have become available in Ghana, West Africa, and have moved the timing of intervention from the late stage to earlier stages in the disease process. CXL is able to stabilize an ectatic cornea detected earlier [12]. However, there are no data indicating the discriminating parameters for forme fruste keratoconus and myopic astigmatism in the Ghanaian population or indicating the continued need for this service. Additionally, the country faces significant challenges in corneal transplantation due to the absence of an established eye bank infrastructure and legislation regarding organ/tissue donations. This shortage of corneal tissues has resulted in a substantial backlog of cases requiring corneal transplants, leaving many individuals affected by corneal blindness without access to essential sight-saving surgeries By leveraging advanced imaging techniques and parameters to differentiate between forme fruste keratoconus and normal cornea, we seek to contribute to the early diagnosis and management of corneal disorders in Ghana.

## Materials and methods

The present investigation conducted a retrospective analysis of clinical records from the Eye Centre of the Komfo Anokye Teaching Hospital in Kumasi - Ghana, encompassing a cohort of 196 eyes from 105 patients with myopic refractive errors. Records were reviewed from July 2019 to August 2021 from the Pentacam HR database. The distribution of study eyes is as follows:

Moderate-High Myopic astigmatism: Age range of 18 to 30 years old, Sphero-cylindrical refractive errors with spherical components >= -2.50D, corrected visual acuity > = 6/18 (Snellen fraction); no contact lens use within 1 month of the examination and a complete clinical data.

Forme fruste keratoconus: Age range of 18 to 30 years old; Sphero-cylindrical refractive errors with spherical components >= -2.50D, corrected visual acuity > = 6/18 (Snellen fraction); no slit lamp findings (no Stromal thinning, Fleischer’s ring, Vogt’s striae, Descemet’s breaks, Apical scars or Subepithelial fibrosis), minor/suspicious topographic keratoconus signs (mild Asymmetric Bow-tie or without skewed axis) or clinical keratoconus in the fellow eye; no contact lens use within 1 month of the examination and a complete clinical data.

Exclusion Criteria: Patients with chronic inflammation of the ocular surface, uveitis, other ocular diseases, or a history of ocular trauma or eye surgery.

Participant’s spherical refractive error and total astigmatism were determined objectively using an Autorefractometer (Humphery^®^ by Carl Zeiss Meditec, Germany) and subjectively by the Maximum plus to Maximum Visual Acuity method at 6 m. Participants who had sphero-cylindrical refractive errors with spherical components greater than − 2.50D were considered to have moderate myopic astigmatism and those with refractive errors greater than − 6.00D were considered to have high myopic astigmatism.

Participants’ topographic and tomographic data were extracted from the Pentacam HR (Typ 70,900 ©Oculus 2013, Oculus Optikgeräte GmbH, Germany). Only scans in which the Pentacam “quality specification” (QS) function determined as “OK” were included for analysis. The following pentacam variables were collected: Index of Surface Variance (ISV), Index of Vertical Asymmetry (IVA), Keratoconus Index (KI), Central Keratoconus Index (CKI), Index of Height Asymmetry (IHA), Index of Height Decentration (IHD), Belin–Ambrósio Enhanced Ectasia Total Deviation Index (BAD-D), Maximum Keratometry from the Anterior Corneal Surface (Kmax), Minimum Corneal Thickness (Ctmin), y cordinate of the Thinnest Corneal Locale (y cordinate), the Posterior Corneal Asphericity (Q(Post.)), the Elevation of the Front Surface at the Thinnest Location (F.Ele.Th), the Elevation of the Back Surface at the Thinnest Location (B.Ele.Th), Minimum Pachymetric Progression Index (RPImin), Maximum Pachymetric Progression Index (RPImax), Average Pachymetric Progression Index (RPIavg) and Maximum Ambrósio Relational Thickness (ARTmax). This study strictly adhered to the principles of the Declaration of Helsinki and was approved by the Committee on human research publication & Ethics (CHRPE) of the Kwame Nkrumah University of Science and Technology. Informed consent was obtained from all participants.

### Data Analysis

Statistical analysis was conducted using IBM SPSS version 23.0 and MedCalc version 22.009 (MedCalc software). To assess normality in the corneal parameters, the Kolmogorov-Smirnov test was employed. The results indicated a normal distribution across parameters, allowing for the use of the independent t-test for intergroup comparisons. A significance level of *p* < 0.05 was considered statistically significant. ROC curves were generated for all parameters to determine optimal cut-off values for maximizing sensitivity and specificity in diagnosing forme fruste keratoconus. Optimum cutoff levels were determined using the Youden index (J) J= (sensitivity + specificity–1) [13]. The parameter value with the maximum Youden index was used as the cut-off value. The area under the curve (AUC) was calculated to assess the overall predictive accuracy. Pairwise comparisons of ROC curves were performed using the DeLong method to detect significant differences between parameter areas (*p* < 0.05).

When selecting variables for inclusion in the binary logistic regression model, a critical criterion was established based on the area under the curve (AUC) obtained from receiver operating characteristic ROC curve analysis. An AUC > 75% (Table 2) was chosen as the cut-off for variable selection, guided by the need to prioritize variables with robust discriminatory power in distinguishing between groups.

Using the forward stepwise entry method with Pentacam variables, Step 1 of the model included variables with an AUC > 75 from the ROC curve analysis. In Step 2, the definitive model incorporated statistically significant variables identified in Step 1. Variance inflation factor (VIF) calculations were used to assess collinearity among variables. The goodness-of-fit of the models was evaluated using the Hosmer-Lemeshow test. Additionally, a classification table was constructed, and an ROC curve was plotted for the binary logistic regression model.

## Results

This study compared 196 eyes from 105 patients divided into two groups: 156 eyes from 79 patients with myopic astigmatism (mean age of 27.2 ± 5.1 years) and 40 eyes from 26 patients with forme fruste keratoconus (mean age of 22.4 ± 3.1 years).There was no statistically significant difference in age distribution between the groups (*p* > 0.05).

Table 1 shows the comparative analysis between eyes with forme fruste and myopic astigmatism. There were statistically significant differences in all pentacam variables (*p* < 0.05) except for the index of height decentration (*p* = 0.66) and the y- cordinate of the thinnest corneal locale (*p* = 0.14). Table 2 shows the results of the ROC curve analysis between eyes with forme fruste and eyes with myopic astigmatism and the cut-off points and corresponding sensitivity and specificity values. The AUC was acceptable (AUC > 0.7) for the ISV, IVA, KI, IHD, BAD_D, Q(Post.), F.Ele.Th, B.Ele.Th, RPImax, RPIavg, ARTmax. In discriminating forme fruste from myopic astigmats, the BAD_D had the highest AUC (AUC = 0.947), followed by the ISV (AUC = 0.906) and then the B.Ele.Th (AUC = 0.897). In discriminating between the two groups, a BAD_D value of 1.57 had the highest sensitivity and specificity, followed by an ISV value of 20.0 and then a B.El.Th value of 5.0. A KI of 1.04 had a sensitivity of 55.0 and a specificity of 89.10. Kmax at a cut-off of 47.0 had a sensitivity of 37.50 and a specificity of 91.03.

**Table 1:** Myopic astigmats versus forme fruste Keratoconus: comparison of pentacam variables

					95% Confidence Interval		
		Mean	Std. Dev.	Std. Error	Lower Bound	Upper Bound	Sig.
ISV	Myopic Astg.	17.61	5.77	0.45	18.86	20.46	0.00
	forme fruste	27.67	6.11	0.97	26.57	28.29	
IVA	Myopic Astg.	0.13	0.05	0.00	0.12	0.14	0.00
	forme fruste	0.19	0.06	0.01	0.18	0.20	
KI	Myopic Astg.	1.02	0.02	0.00	1.016	1.024	0.00
	forme fruste	1.04	0.03	0.00	1.03	1.05	
IHA	Myopic Astg.	5.43	4.64	0.37	4.74	6.12	0.00
	forme fruste	8.28	5.61	0.89	7.75	8.81	
IHD	Myopic Astg.	0.05	0.54	0.04	0.00	0.47	0.66
	forme fruste	0.01	0.01	0.00	0.00	0.05	
BAD_D	Myopic Astg.	0.88	0.53	0.04	0.98	1.04	0.00
	forme fruste	1.98	0.44	0.07	1.90	2.25	
Kmax	Myopic Astg.	44.80	1.76	0.14	41.35	48.25	0.00
	forme fruste	45.97	1.61	0.25	43.80	48.15	
Ctmin	Myopic Astg.	532.99	33.10	2.65	499.07	566.91	0.00
	forme fruste	510.20	26.19	4.14	483.76	536.64	
y cordinate	Myopic Astg.	-0.54	0.37	0.03	-0.83	-0.25	0.14
	forme fruste	-0.63	0.27	0.04	-0.93	-0.33	
Q (Post.)	Myopic Astg.	-0.32	0.12	0.01	-0.48	-0.16	0.03
	forme fruste	-1.27	5.47	0.87	-2.03	0.49	
F.Ele.Th	Myopic Astg.	2.34	1.59	0.13	0.22	4.46	0.00
	forme fruste	4.35	2.36	0.37	1.70	6.00	
B.Ele.Th	Myopic Astg.	4.04	3.14	0.25	0.97	7.19	0.00
	forme fruste	10.88	4.47	0.71	4.76	14.20	
RPImin	Myopic Astg.	0.71	0.16	0.01	0.52	0.90	0.00
	forme fruste	0.83	0.22	0.04	0.64	1.02	
RPImax	Myopic Astg.	1.92	8.07	0.65	0.65	3.20	0.00
	forme fruste	1.74	1.84	0.29	1.17	2.31	
RPIavg	Myopic Astg.	1.00	0.13	0.01	0.75	1.25	0.00
	forme fruste	1.13	0.13	0.02	0.88	1.38	
ARTmax	Myopic Astg.	427.29	72.17	5.78	354.74	499.84	0.00
	forme fruste	0.358.88	52.15	8.25	308.74	409.02	
CKI	Myopic Astg.	1.01	0.01	0.00	0.99	1.03	0.00
	forme fruste	1.01	0.00	0.00	-	-	

**Table 2:** Receiver-operating characteristic curve analysis for myopic astigmatism versus Forme fruste keratoconus

Parameter	AUC	95% CI	SE	Cut-off	Sensitivity	Specificity
ISV	0.906	0.856–0.943	0.021	20.0	97.50	77.56
IVA	0.796	0.733–0.850	0.035	0.13	90.00	59.62
KI	0.755	0.689–0.814	0.047	1.04	55.00	89.10
IHA	0.672	0.601–0.737	0.046	6.20	65.00	64.74
IHD	0.726	0.640–0.812	0.044	0.013	62.50	76.92
BAD_D	0.947	0.905–0.974	0.015	1.57	92.50	91.67
Kmax	0.689	0.601–0.778	0.045	47.0	37.50	91.03
Ctmin	0.292	0.207–0.377	0.043	521	70.00	61.54
y cordinate	0.39	0.294–0.485	0.049	0.53	75.00	48.72
Q (Post.)	0.734	0.666–0.794	0.041	-0.37	72.50	71.15
F.Ele.Th	0.791	0.703–0.880	0.045	3.00	75.00	76.28
B.Ele.Th	0.897	0.845–0.935	0.028	5.00	92.50	76.28
RPImin	0.684	0.589–0.779	0.048	0.76	62.50	67.95
RPImax	0.758	0.692–0.816	0.041	1.41	57.50	80.13
RPIavg	0.782	0.718–0.838	0.039	1.01	87.50	58.97
ARTmax	0.772	0.707–0.829	0.038	392	72.50	69.87
CKI	0.642	0.555–0.729	0.044	1.00	95.0	30.77

AUC, area under the curve; SE, standard error

In the logistic regression, the dependent variable was the presence of forme fruste vs. myopic astigmatism. The variables entered into the first step of the model were ISV, IVA, BAD_D, B.Ele.Th, Artmax, AvgProg, MaxProg, F.Ele.Th and KI (see Table 3). The final model comprised variables that showed statistically significant differences from the first step of the model between the two groups; ISV, BAD_D, B.Ele.Th and KI (*p* < 0.05) between the two groups (see Table 4). A hypothesis contrast test was performed using the Wald test to compare both models. There was no statistically significant difference in the coefficient estimates (*p* > 0.05) for the predictor variables between the two models and as such the model with the simpler variables (Model 2) was chosen. The final model was expressed in the form of an algorithm: Logit (*p) =* -55.92 + 0.23(ISV) + 6.19(BAD_D) + 0.310(B.Ele.Th) + 35.94(KI).

**Table 3:** Variables in the equation in step 1 of the Binary Logistic regression

							95% C.I. for EXP(B)	
	B	S.E	Wald	df	Sig.	Exp(B)	Lower	Upper
ISV	0.200	0.101	3.922	1	0.048*	1.221	1.002	1.489
IVA	12.817	11.691	1.202	1	0.273	368559.283	0.00	3.293E + 15
BAD_D	5.882	2.137	7.576	1	0.006*	358.487	5.439	23629.670
B.Ele.Th	0.355	0.117	9.216	1	0.002*	1.426	1.134	1.793
Artmax	-0.007	0.015	0.229	1	0.633	0.993	0.963	1.023
AvgProg	-0.487	6.156	0.006	1	0.937	0.615	0.00	106810.628
MaxProg	-0.289	0.780	0.137	1	0.711	0.749	0.162	3.456
F.Ele.Th	-0.322	0.280	1.323	1	0.250	0.725	0.419	1.254
KI	44.839	18.630	5.793	1	0.016*	2.975E + 19	4128.408	2.143E + 35
Constant	-61.473	21.817	7.939	1	0.005	-	-	-

*p < 0.05; B, coefficient; S.E, Standard error; Wald, forward selection; Exp(B), odds ratio

**Table 4:** Proposed logistic binary logistic model (model 2)

							95% C.I for EXP(B)		
	B	S.E.	Wald	df	Sig.	Exp(B)	Lower	Upper	VIF
ISV	0.226	0.085	7.058	1	0.008	1.253	1.061	1.480	1.68
BAD_D	6.190	1.611	14.759	1	0.00	487.726	20.737	11471.136	1.73
Belevation	0.310	0.102	9.261	1	0.002	1.364	1.117	1.666	1.75
KI	35.944	16.568	4.707	1	0.030	4.078E + 15	32.181	5.167E + 29	1.13
Constant	-55.921	18.381	9.256	1	0.002	-	-	-	

VIF, Variance inflation factor

**Fig. 1:**
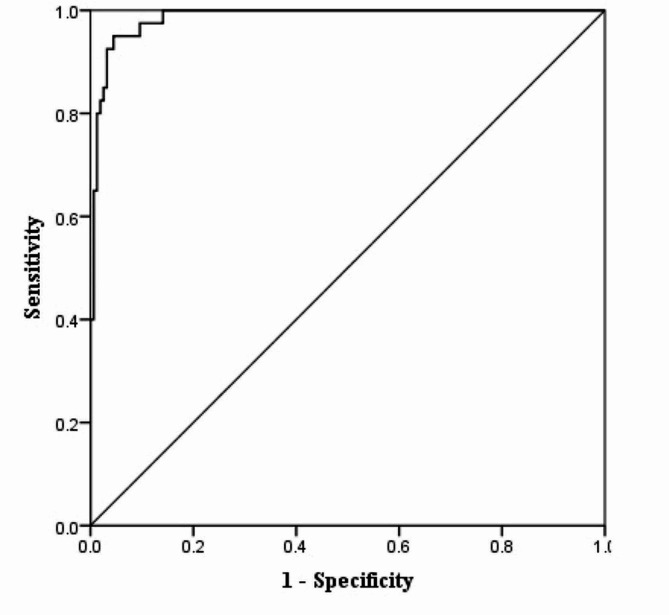
ROC curve for the logistic regression model for the forme fruste keratoconus

The classification table of the proposed model is presented in Table 5. The sensitivity was 92.5%, the specificity was 96.8%, the accuracy was 95.9%, and the positive predictive value was 88.1%. The AUC of the ROC curve for the proposed model was 0.986 (95% CI 0.973–0.999) (see Fig. 1).

**Table 5:** Logistic binary regression model: classification table

	Predicted		
Observed	Normal	Forme fruste	Percentage correct
Normal	151	5	96.8
Forme fruste	3	37	92.5
Overall Percentage			95.9

## Discussion

The primary objective of this study was to evaluate the diagnostic potential of scheimpflug photography with the Pentacam system for distinguishing between moderate-to-high myopic astigmatism and forme fruste keratoconus. The comparison between moderate to high myopic astigmatism and forme fruste keratoconus revealed statistically significant differences in most pentacam variables. This suggests that these variables are sensitive enough to detect variations between the groups, which is a crucial finding in understanding the differences in corneal characteristics between these conditions. However, it is important to note that statistical significance alone does not necessarily imply clinical significance or its diagnostic utility. Binary logistic regression analysis refined the diagnostic model by selecting ISV, BAD_D, B.Ele.Th, and KI based on their significant differences between groups (*p* < 0.05). The final model achieved high sensitivity, specificity, accuracy, and a positive predictive value.

The BAD is a comprehensive display that enables a global view of the tomographic structure of the cornea through the combination of elevation and pachymetric data. Deviation of normality values were implemented for the front and back enhanced elevations, thinnest value, pachymetric distribution and vertical displacement of the thinnest in relation to the apex point of the cornea. The final “D” is calculated based on a regression analysis that weights each parameter differently [14]. The ability of the BAD_D to discriminate keratoconus has been reported in several studies [7, 15, 16]. In instances of detecting subclinical/ forme fruste keratoconus, the BAD_D in addition to other parameters, has proven to some extent to detect the condition [17–22].

Hashemi et al. [18] and our study both underscore the significance of Belin/Ambrosio Deviation Display (BAD_D) and the Index of Surface Variance (ISV) in diagnosing subclinical keratoconus. Hashemi et al. reported that BAD_D, with a sensitivity of 81.1% and specificity of 73.2% at a cut-off of 1.54 and an AUC of 0.86, showed promising diagnostic accuracy. Similarly, in our study population, BAD_D exhibited high sensitivity (92.5%) and specificity (91.67%) at a cut-off of 1.57. This consistency in the performance of BAD_D across studies highlights its effectiveness as a discriminating parameter between keratoconus subtypes and myopic astigmatism. Moreover, the ISV, as noted in both studies, also demonstrated notable diagnostic capability. Hashemi et al. reported an ISV sensitivity of 74.5% and specificity of 61.8% at a cut-off of 22. In our study, the ISV showed a sensitivity of 97.5% and specificity of 77.56% at a cut-off of 20. These findings suggest that the ISV, alongside BAD_D, plays a crucial role in improving early keratoconus detection and guiding clinical decisions. The higher sensitivity observed in our study for both BAD_D and ISV could be attributed to various factors such as differences in sample characteristics. In the study by Hashemi et al., the keratoconus group had been diagnosed in the clinic after which the severity of the condition was grouped according to the classification by McMahon et al. [23]. This may imply that, the subclinical group had more pronounced corneal characteristics than did the patients in the current study.

Similarly, Vazquez et al. [19] reported the efficacy of the Pentacam indices in differentiating between topographically normal patients and those with subclinical keratoconus. Notably, BAD_D emerged as a standout parameter, exhibiting a sensitivity of 89.2% and a specificity of 82.3% at a cut-off of 1.61, highlighting its effectiveness in diagnosing subclinical keratoconus. This finding was consistent with the current study.

Nicula et al. [20] investigated the efficacy of Pentacam indices in differentiating clinical and subclinical keratoconus from normal eyes. Their study revealed that specific Pentacam indices, including IVA and BAD_D, demonstrated superior performance in identifying subclinical keratoconus patients. BAD_D, in particular, emerged as a crucial parameter with high discriminatory ability, emphasizing its significance in the diagnosis of keratoconus, especially its subclinical manifestation.

In addition to the BAD_D, we also considered the ISV indices obtained from the corneal curvature which remained in the final model for detecting forme fruste cases. The index of surface variance (ISV) is the deviation of the corneal radius from the mean value (it reflects the regularity of the corneal surface). The ISV may be useful for tracking keratoconus progression [18, 24–26].

In a study by Wang et al. [24] on high-risk allergic conjunctivitis patients, the ISV showed a significant correlation with changes in corneal epithelial thickness, particularly in keratoconus-susceptible individuals. Similarly, Kanellopoulos and Asimellis [25] investigated epithelial thickness in keratoconus and found that the ISV was a strong indicator of topographic epithelial thickness changes. Their earlier study in 2013 [26] also highlighted the role of the ISV in classifying keratoconus severity and progression. These findings align with our study, where the ISV played a crucial role in predicting forme fruste keratoconus, emphasizing its relevance as an early indicator of keratoconus-related changes.

The posterior corneal surface has been reported by several studies to be a good indicator for early keratoconus [22, 27, 28]. In the study by Somali et al. [29], The Back Elevation at the thinnest corneal locale (B.Ele.Th) remained a top parameter in differentiating subclinical keratoconus from normal cases. Alongside BAD_D, total higher-order aberrations, average pachymetric progression index and Ambrosio relational thickness deviation showed significant discriminatory ability. Similarly, Vlasak et al. [30] identified the B.Ele.Th as the most effective parameter for detecting subclinical keratoconus within the Czech population. The Keratoconus Index is the ratio between the mean radius in the upper and lower segments. In their 2018 study among young Caucasians, Huseynli and Abdulaliyeva reported that several pentacam parameters showed good predictive accuracy in detecting subclinical keratoconus, although the difference was less pronounced than that in definite keratoconus eyes. These indices included pachymetric progression indices, the ISV and the KI [31]. This finding is consistent with the current study.

It is noteworthy that despite overlapping parameters, our examination did not reveal multiple studies utilizing identical parameters in the predictive model for forme fruste/subclinical keratoconus. This highlights the complexity of diagnosing forme fruste keratoconus with differences in parameters likely arising from variations in study populations or definitions of forme fruste keratoconus. Our study stands out by highlighting critical differences in Pentacam parameter cut-offs for diagnosing forme fruste keratoconus in African populations. For instance, the cut-offs for significant parameters such as BAD_D, ISV, B.Ele.Th, and KI in our study were different from those reported in other ethnic groups. These differences underscore the importance of population-specific diagnostic criteria and provide valuable contributions to the diagnostic process for forme fruste keratoconus, particularly within Ghanaian population.

Several limitations must be acknowledged, including potential selection bias due to the retrospective nature of the study, a relatively small sample size from a single center, and the absence of longitudinal follow-up data. Additionally, environmental and genetic factors were not fully explored, which may affect diagnostic performance. While the AUC was used for variable selection in the logistic regression model, other statistical approaches or machine learning algorithms could be explored to identify optimal combinations of variables for improved predictive accuracy. External validation of the predictive model using independent datasets is essential to confirm its generalizability and reliability across different patient populations and clinical settings. The determination of cut-off values for Pentacam variables was based on statistical analysis, and future studies could investigate the clinical relevance and optimal diagnostic thresholds of these variables through correlation with clinical outcomes and long-term follow-up data.

## Conclusion

The combined evaluation of BAD_D, ISV, B.Ele.Th, and KI prove to be beneficial for identifying cases of forme fruste keratoconus in an African population. The suggested cut-off points in our research demonstrated acceptable sensitivity and specificity, indicating their potential clinical utility. However, additional studies focusing on refining these cut-off values and assessing their repeatability across various age and sex groups are warranted to further enhance diagnostic accuracy.

## Data Availability

No datasets were generated or analysed during the current study.
